# O6.6. LACK OF ANTIPSYCHOTIC MEDICATION EFFECTS ON WHITE MATTER MICROSTRUCTURE IN SCHIZOPHRENIA

**DOI:** 10.1093/schbul/sby015.227

**Published:** 2018-04-01

**Authors:** Nina Kraguljac, Anthony Thomas, Frank Skidmore, David White, Adrienne Lahti

**Affiliations:** University of Alabama at Birmingham

## Abstract

**Background:**

A number of studies have reported decreased white matter integrity in patients with schizophrenia, but little is known about the relationship between white matter integrity and antipsychotic medications.

**Methods:**

We enrolled 42 unmedicated patients (thirty were medication-naïve) with schizophrenia in a longitudinal trial with risperidone. Symptom severity was assessed with the Brief Psychiatric Rating Scale (BPRS). We obtained diffusion weighted images before medication was started, and after six weeks of treatment. Healthy controls matched 1:1 on age, gender, and parental socioeconomic status were also scanned twice six weeks apart. 30 diffusion sampling directions spanning the whole sphere were acquired twice and concatenated (in plane resolution 2.2mm, slice thickness 2.2mm, b-value 1000 s/mm2, 5 b0 images). After visual inspection of raw images we used TORTOISE for correction of bulk motion, eddy currents and susceptibility artifacts using a single interpolation step in DIFF_PREP. For each dataset, the first B0 image was selected as the reference for registration. Prior to registration, diffusion weighted and structural images were upsampled at a factor two and smoothed with a Perona-Malik anisotropic edge favoring gradient based filter to compute the transformations from moving to fixed images. After computation of transformations, original images were used to create the registered images. Bspline correction was done with the T2 weighted image (approximated from a T1 image using AFNI’s FATCAT). Diffusion and structural images were resampled to 1.5mm isotropic voxels. Gradient tables were rotated along with motion correction. To obtain a summary measure of motion, the root-mean-square (RMS) was calculated both for absolute (RMSabs) and relative (RMSrel) movement. Datasets with RMSabs of greater than the voxel edge length were excluded from further analysis. Tensors were computed with DIFF_CALC using a linear fitting algorithm. To spatially normalize diffusion images to the Illinois Institute of Technology atlas (IIT2) space, we implemented an optimized non-linear image registration using a modified version of 3dQwarp in AFNI. The warping optimization implements an iterative refinement, where an input image is repeatedly processed through an optimizer in smaller and smaller patches, incorporating convergence criteria at each patch level to better resolve artifacts, with a final patch size of 3x 5x 3 mm. To assess whole brain voxel-wise group differences and changes over time in scalar indices used AFNI’s 3dttest++ (age, sex, and RMSrel as covariates) with clustsim, a bootstrapping method used to correct for multiple comparisons.

**Results:**

Mean age of patients was 26.62 years, 62% of subjects were male. Of the 42 patients included here, 33 completed the study. BPRS total scores decreased significantly after six weeks of treatment, average risperidone dose at that time was 3.73+/-1.72mg. Fractional anisotropy (FA) was significantly decreased in a small area of the medial temporal lobe and mean diffusivity (MD) was significantly increased in the hippocampal part of the cingulum in unmedicated patients (n=40) compared to healthy controls (n=41). Longitudinal analyses showed no changes in FA, MD, RD or white matter macrostructure in healthy controls over time, and no changes in patients after six weeks of treatment with risperidone.

**Discussion:**

With state of the art data-processing methods we only found small areas of white matter integrity deficits in our predominantly medication-naïve patients. This is consistent with prior reports of limited white matter alterations at disease onset that may progress with illness duration. Our data suggests that a short-term course of antipsychotic medication may not alter white matter microstructure, but studies with longer follow up durations will be important to determine long term effects of antipsychotic medications.

